# Hemodynamic Characterization of Peripheral Arterio-Venous Malformations Using Rapid Contrast-Enhanced MR Imaging: An In Vitro and In Vivo Study

**DOI:** 10.1007/s10439-025-03766-3

**Published:** 2025-06-13

**Authors:** Camilla Giulia Calastra, Marika Bono, Aloma Blanch Granada, Aleksandra Tuleja, Sarah Maike Bernhard, Vanessa Diaz-Zuccarini, Stavroula Balabani, Dominik Obrist, Hendrik von Tengg-Kobligk, Bernd Jung

**Affiliations:** 1 Department of Diagnostic, Interventional and Pediatric Radiology (DIPR), Inselspital, Bern University Hospital, University of Bern, Bern, Switzerland; 2 ARTORG Center for Biomedical Engineering Research, University of Bern, Bern, Switzerland; 3 Department of Mechanical Engineering, University College London, Torrington Place, London, WC1E 7JE UK; 4 Division of Angiology, Swiss Cardiovascular Center, Inselspital, Bern University Hospital, University of Bern, Bern, Switzerland; 5 Wellcome/EPSRC Centre for Interventional and Surgical Sciences (WEISS), Department of Medical Physics and Biomedical Engineering, University College London, 43-45 Foley Street, London, W1W 7TS UK; 6 Translational Imaging Center (TIC), Swiss Institute for Translational and Entrepreneurial Medicine, Bern, Switzerland; 7 Experimental Radiology, Department of BioMedical Research, University of Bern, Bern, Switzerland

**Keywords:** Vascular malformations, Yakes AVMs classification, MR imaging, Validation, CE-trMRA

## Abstract

**Purpose:**

Peripheral arterio-venous malformations (pAVMs) are vascular defects often requiring extensive medical treatment. To improve disease management, hemodynamic markers based on 2D Digital Subtraction Angiography (DSA) data were previously defined to classify pAVMs. However, DSA offers only 2D information, involves ionizing radiation, and requires intra-arterial intervention. We hypothesized that pAVMs could be classified with the same approach with 3D dynamic contrast-enhanced MR-based data. To this end, the present work aims to develop a computational classification system for pAVMs using 3D dynamic contrast-enhanced MR-based data.

**Methods:**

A pAVM phantom was imaged using both DSA and MRI to validate the methodology, which was then applied to 10 MR-based in vivo datasets. A semi-automated vessel detection algorithm, based on the standard deviation of each voxel or pixel in time, was used. Classification was performed by identifying the time of arrival (CA_ToA_) of contrast agent (CA) and the maximum time derivative of the CA transport in each pixel or voxel (CA_si_).

**Results:**

Normalized CA_ToA_ and CA_si_ histograms showed no significant difference between in vitro DSA and MRI (respectively *χ*^2^ = 0.20, *p* = 0.65 and *χ*^2^ = 0.21, *p* = 0.65), validating the methodology to classify pAVMs. CA_ToA_ histograms for type II–IV AVMs derived from in vivo MR-based data aligned with DSA patterns and known hemodynamics. CA_ToA_ histograms of capillary–venulous AVMs were distinct, with non-zero values at later times than other AVM types, representing late venous drainage. Type IV AVMs histograms for CA_si_ were more right-skewed than those derived from types II and III pAVMs.

**Conclusions:**

MR image quality and temporal resolution are sufficient to allow a classification of pAVMs. This classification method has the potential to become a diagnostic tool for the surgical navigation of pAVMs for clinicians.

**Supplementary Information:**

The online version contains supplementary material available at 10.1007/s10439-025-03766-3.

## Introduction

Congenital vascular malformations (CVMs) are localized anomalies arising during the embryonic development of the vascular system [1]. CVMs can occur anywhere in the body and exhibit varying blood flow dynamics. Peripheral arterio-venous malformations (pAVMs), a CVM subtype, are high-flow congenital defects found outside the central nervous system [2] and have a prevalence of approximately 0.03% [3]. pAVMs consist of tangles of abnormal blood vessels where the feeding arteries are directly connected to a venous drainage network, without the interposition of capillaries [3]. These malformations tend to become large and complex, leading to various physical complications including tissue damage due to the steal phenomenon and consecutive ischemia, venous hypertension, excessive tissue growth, and volume overload of the heart.

The gold standard imaging technique for diagnosing and treating pAVMs is dynamic intra-arterial catheter-based 2D digital subtraction angiography, DSA [4, 5], a mere luminography, often supplemented by magnetic resonance (MR) imaging and duplex ultrasound (US). MR Imaging plays a role in the first-time diagnosis [6] by offering a non-invasive imaging modality which delineates the nidus, feeding arteries, and draining veins. It is also used in the planning of the surgical treatment for the 3D localization of the pAVM and for post-therapy follow-up, providing a radiation-free alternative to repeated DSA, especially valuable in younger or recurrent patients [7].

Extensive treatment may be required for pAVMs due to their impact on local tissue structures and the cardiovascular system [8], involving repeated exposure to X-ray radiation and surgical interventions. Additionally, the intricate structure and altered blood flow patterns of pAVMs often make treatment challenging and sometimes unsuccessful [9]. Several classification systems, including that of Yakes et al. [8, 9], have been developed to guide pAVMs treatment based on standardized lesion angioarchitectures. Yakes classification categorizes pAVMs in type I, a direct artery-to-vein shunt; type II, multiple arteries connected to vein-venular structures (*nidus*); type III, multiple arteries lead to a single aneurysmal vein (type IIIa) or multiple dilated veins (type IIIb); and type IV, microfistulous connections with parallel capillary perfusion. In addition, recently, a new subgroup of microfistular pAVMs, the capillary–venulous arterio-venous (CV-AVM) has been proposed, composed of fistulous paths on the venous half of capillaries and dilated draining venules [10, 11].

However, these classifications overlook hemodynamic changes induced by patient-specific lesion characteristics. Quantifying patient-specific hemodynamic changes may be beneficial for classification and for more personalised treatment planning.

So far, medical image processing techniques integrated with engineering tools have shown promise for predicting vascular hemodynamics [12–14], leading to the identification of hemodynamic biomarkers in several vascular diseases, including aortic dissection [15–18], coronary artery disease [19], peripheral arterial disease [20], and cerebral aneurysms [21]. Studies have investigated hemodynamic patterns and treatment strategies through computational modeling [22–24] and treatment outcomes with the use of machine learning [25, 26] for cerebral AVMs (cAVMs). Even if medical imaging processing with machine learning is increasingly applied to vascular problems, we are not aware of such studies in the context of pAVMs.

Recently, Frey et al. [27] used the information on the transport of contrast agent (CA) as captured by dynamic DSA to define markers to characterize and classify pAVMs by their distinct hemodynamic appearance.

To further validate and develop such hemodynamic markers, artificial vascular models offer a powerful platform for investigating patient-specific flow dynamics under controlled conditions.

These models have been extensively used in cardiovascular research for exploring blood flow mechanics [28–30], pre-surgical planning [31–33], and validating imaging techniques [33–35].

However, due to the structural complexity and small vessel dimensions of pAVMs, their application in this context is still in its early stages. Recent works have demonstrated the feasibility of constructing physiologically realistic 3D-printed models of both cAVMs [35] and pAVMs [30, 36], enabling flow characterization in vitro*.*

We hypothesize that state-of-the-art MR Imaging acceleration techniques, such as Parallel Imaging [37–42] and Compressed Sensing [43–46], in combination with image processing may enable the classification of pAVMs using the hemodynamic markers described in [27].

To this end, the present work aims to develop a computational classification system for pAVMs using non-invasive, ionizing radiation-free MR Imaging. To achieve this goal, the study is divided into two main parts:

- Validation of the hemodynamic markers in a controlled in vitro experimental set-up in DSA and MR environments
- Application of the image processing algorithm to in vivo MR Imaging datasets.

## Materials and Methods

### In Vitro Data

To verify the ability of MR Imaging to reproduce the hemodynamic markers originally defined in DSA [27], a direct comparison was conducted between the two modalities with the same phantom under two separate experimental conditions adapted to the respective imaging modalities.

**Fig. 1:**
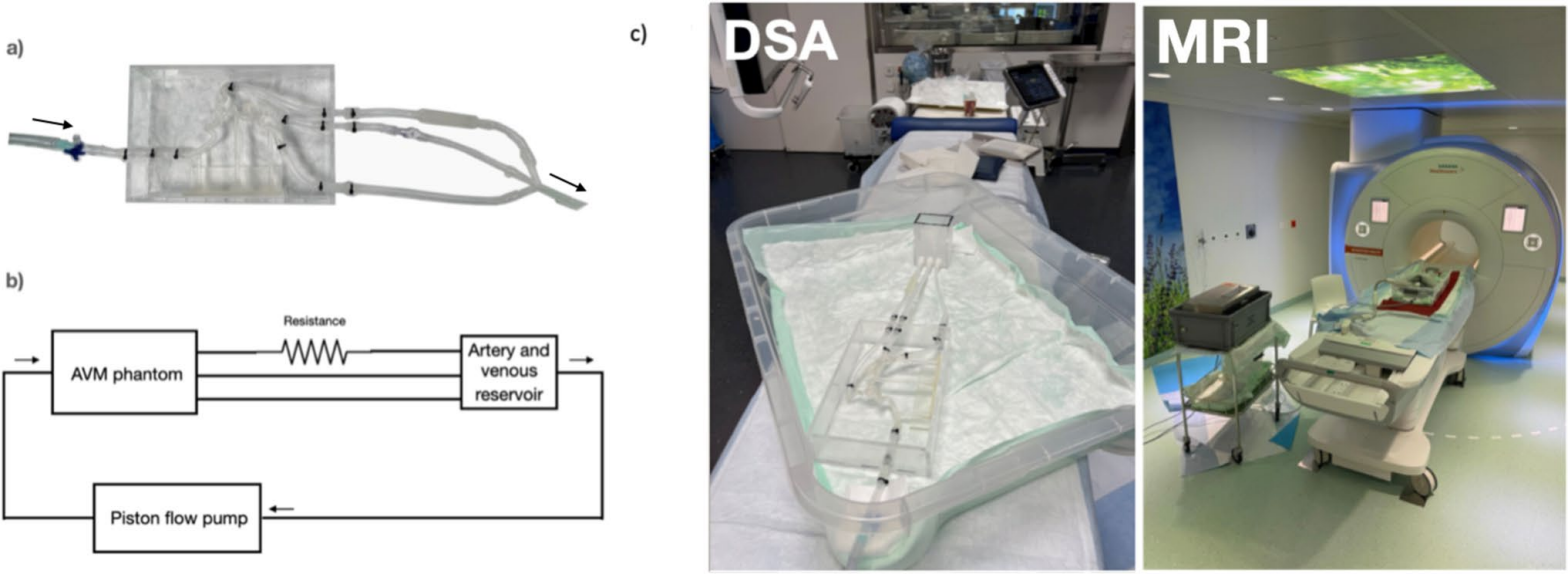
**a** 3D-printed pAVM phantom. **b** Diagram of the experimental set-up. The arrow represents the direction of flow. **c** Experimental set-up in interventional suite and MR environment

A rigid replica of a type III neck pAVM from a 47-year-old man was fabricated using clear resin via stereolithography, achieving a maximum resolution of 25 µm with a Formlabs Form 3 SLA 3D printer (Formlabs, USA) [36] (Fig. 1a). The patient-specific geometry of the pAVM was segmented from a CT scan as described by Franzetti et al [30]. The reconstructed geometry preserved the spatial configuration of the vascular network, comprising a 3.8 mm diameter feeding artery, a 2 mm arterial outflow, a nidus measuring approximately 16 × 12 × 9 mm, and two draining veins with diameters of 1.6 mm and 2.3 mm, respectively. The nidus region was modeled as an idealized porous lattice that induces the same pressure drop as the in vivo case, as estimated from the computational fluid dynamic studies of the same pAVM [30]. To facilitate integration with the experimental set-up, minimize flow transitions, and prevent leakages, barbed fittings were added to the inlet and outlets [35].

The dataset was acquired as part of an ethical protocol, and data were used after appropriate patient consent (NHS Health Research 94 Authority, ref: 19/SC/0090). The phantom can be compared to an extremity as one artery has been identified as the main feeder and two veins as the main draining veins, which resembles the configuration of a pAVM of a foot or a hand, characterized by one of few feeding arteries and one or few draining veins.

The phantom was connected to an experimental set-up, with a 90:10 water-glycerol mixture (99%, SIGALD, Sigma-Aldrich Chemie GmbH, Buchs) used as the working fluid. The resulting density and viscosity of the fluid were *ρ* = 1024*.*2 kg/m^3^ and *µ* = 1.2744 mPa s. Although this mixture did not match the viscosity and density of blood, it was selected to match the T1 relaxation time of blood at 3 T [47]. To ensure comparability between imaging modalities, the same working fluid was also used for DSA experiments.

A MR-compatible piston pump (CompuFlow 1000 MR, Shelley Medical Imaging Technologies, Simutec, Ontario, Canada) delivered a patient-specific flow curve. The average inlet flow rate was estimated using patient-specific in vivo DSA data, and a common carotid artery waveform [48] was scaled to match this average flow rate, serving as the inlet boundary condition. Under these conditions, the flow remains laminar and is representative of the blood flow in the studied vasculature. A summary of the experimental parameters for DSA and MR Imaging is reported in Table 1.

**Table 1:** Summary of experimental parameters for DSA and MR GRASP

Parameter	DSA	GRASP
Flow waveform frequency (Hz)	1.095	0.36
Average flow rate, *Q*_avg_ (mL/s)	1.06	0.33
Maximum flow rate, *Q*_max_ (mL/s)	4.82	1.59
Average Reynolds number	289.65	90.91
Womersley number (*α*)	3.49	1.87
Injection distance to phantom (m)	~ 0.5	~ 3
Contrast agent (CA)	100% Iodine CA (300 mg/ml, Bracco Suisse SA, Switzerland, Cadempino)	Gadolinium-based CA (GBCA) (Gadovist 1.0 M, Bayer, Switzerland AG, Zurich)
CA injection rate (mL/s)	5	4
CA injection duration (s)	4	2
CA injection volume (mL)	20	8
Tubing length (pump to phantom)	~ 1.0 m	~ 5.0 m

In the interventional suite, tubes between the flow pump and the phantom were approximately 1 m long; in the MR scanner suite, tubes were approximately 5 m long to maintain the safety distance between pump unit and scanner. Similarly, the CA injection for DSA was performed approximately 50 cm away from the AVM phantom, whereas in the MR suite approximately 3 m away to mimic clinical practice. The amount of volume injected was chosen to match the in vivo conditions. For MR Imaging, the CA dosage was determined to be 10% (mL) of the patient’s body weight (kg) (assumed 80 kg), with a fixed flow rate of *Q* = 4 mL/s, as for a clinical in-house protocol. A summary of the CAs used and injection parameters is found in Table 1.

Dynamic DSA was performed using a clinical standard angio suite (Azurion, Philips, Einthoven, NL). 3D contrast-enhanced time-resolved magnetic resonance angiography (CE-trMRA) was performed on a clinical 3 T MR scanner (Magnetom Vida, Siemens, Erlangen, Germany) using a Golden-Angle Radial Sparse Parallel (GRASP) sequence. A diagram of the experimental set-up is shown in Fig. 1b, and images of the DSA and MR Imaging setups are provided in Fig. 1c. The acquisition parameters are summarized in Table 2. A receiver 4-element flexible coil was positioned on top of the phantom to be combined with and a 34-element spine coil within the patient MR table.

**Table 2:** Acquisition parameters for in vitro DSA and MR GRASP sequence

Parameter	DSA	GRASP
Slice thickness (mm)	N/A	1.4
Temporal resolution (s)	0.125	1.1
*T*_R_ (ms)	N/A	3.62
*T*_E_ (ms)	N/A	1.39
Flip angle (°)	N/A	25
Scan duration (s)	16.125	141
Spatial resolution (mm)	0.15 × 0.15	1.35 × 1.35
Field of view (FOV) (mm^2^)	N/A	259 × 259
Number of projections/slices	1	32
Matrix size	1016 × 1016	192 × 192

### In Vivo Data

In this study, 10 CE-trMRA datasets of human in vivo AVMs were analyzed. The cohort comprises patients who were treated in our hospital and presented with a pAVM in the extremities (*n* = 8 with hand AVM, *n* = 2 with foot AVM) that underwent a dedicated MR examination, including a CE-trMRA at 3 T. All MR imaging data were available at the local Picture Archiving and Communication System (PACS) at our institution. MR imaging data were acquired in three Swiss hospitals, between 2017 and 2023.

The study received approval from the Ethics Committee of the Canton of Bern (IRB Number 2017-01960) and all patients gave their written informed consent prior to the MR Imaging examination.

By concentrating solely on the extremities, it was possible to make direct lesion comparisons between patients. Details on patients' demographics and image acquisition parameters are found in Table 3.

**Table 3:** pAVM types in hand and foot

ID	Type	Body region	Temp res. (s)	Sp. res. (mm)	Sl. thickness (mm)	Acquisition scheme
ID001	II	Hand	1.100	0.741 × 0.741	2.600	Cartesian
ID002	II	Hand	1.100	0.740 × 0.740	2.600	Cartesian
ID003	II	Hand	4.900	1.086 × 0.717	1.100	Cartesian
ID004	IIIB	Hand	1.300	0.741 × 0.741	2.000	Radial
ID005	IIIB	Hand	4.500	0.920 × 1.395	1.100	Cartesian
ID006	IV	Foot	1.400	0.741 × 0.741	2.000	Cartesian
ID008	CV-AVM	Foot	4.600	0.741 × 0.741	2.000	Cartesian
ID009	CV-AVM	Hand	1.400	0.741 × 0.741	2.000	Cartesian
ID012	CV-AVM	Hand	3.300	0.741 × 0.741	2.600	Cartesian
ID020	IV	Hand	1.300	0.741 × 0.741	2.500	Radial

*Type* refers to the pAVM type according to Yakes classification, *Temp. res.* temporal resolution, *Sp. res.* spatial resolution, *Sl. thickness* thickness in the *z*-direction, perpendicular to the imaging acquisition plane. Acquisition scheme refers to the acquisition scheme, Cartesian or Radial

### Image-Processing

#### Preprocessing

The DSA images were first processed using background subtraction; this was done by removing the initial time frame, taken before CA injection, from all subsequent frames. Subsequently both DSA and MR Imaging datasets were organized into a 3D structure for DSA and a 4D structure for CE-trMRA.

Images were cropped to remove irrelevant pixels, artifacts, and overlaid digits. A 1D median filter was used to smooth temporal intensity spikes: a five-time-step window for CE-trMRA and three-time-step for DSA were employed, reflecting differences in spike intensity. A 2D median filter reduced salt and pepper noise. In CE-trMRA, a 2 × 2 voxel filter was used, which was smaller due to lower spatial resolution compared to 6 × 6 pixel filter of DSA. Normalization was carried out across all images within the 3D or 4D intensity volume. A 7–10% noise threshold was applied to balance noise reduction and vessel visualization.

In MR Imaging, a masking procedure was applied by integrating a vessel detection algorithm, which evaluated the standard deviation of intensity values across all voxels over time. A patient-specific standard deviation threshold visually determined and ranging between 2.7 and 3.4, identified voxels exhibiting substantial intensity fluctuations indicative of CA presence.

#### Postprocessing

The hemodynamic markers defined by Frey et al. [27] classify pAVMs based on the information derived from the transport of CA as observed in DSA images.

*Time of Arrival of the Contrast Agent* the time of arrival (ToA) of the CA, denoted as CA_ToA_, is calculated as the time from injection until the signal intensity reaches 10% of the maximum signal intensity value. This estimation accounts for different CA arrival times due to the variability of datasets.

CA_ToA_ reflects how quickly different vascular territories are reached by the CA. From a clinical perspective, early arrival of CA in the venous system may indicate direct arterio-venous shunting—a hallmark of type I AVMs—whereas delayed and more distributed CA arrival is more typical for complex lesions such as type III AVMs or CV-AVM. These features may have diagnostic value, as shorter CA_ToA_ in draining veins reflects faster transit times through low-resistance shunts and may be linked to high-flow lesion.

The CA_ToA_ values estimated from the in vitro CA distributions were normalized by identifying the pixel or voxel with the longest CA arrival and subtracting each voxel or pixel’s CA_ToA_ from this value, and scaling it by the total effective time in which the CA was present, obtaining CA_ToA_^norm^. This process accounts for variations in CA injection timing between MR Imaging and DSA and facilitates direct comparisons.

*Dispersive Slope of the Contrast Agent* the rate of CA increase is determined by calculating the maximum rate of change of the CA transport in each pixel or voxel using a finite difference:

$${\text{CA}}_{\text{si}}=\frac{c\left(i,t+\Delta t\right)-c\left(i,t\right)}{\Delta t}.$$

Here, ∆*t* represents the sampling rate, and *c*(*i*,* t*) is the *i*th pixel/voxel concentration value at time* t.*

It is important to note that the CA is injected at a specific location, leading to dilution where vessels with CA converge with vessels carrying CA-free blood. We performed a pixel- or voxel-wise time-intensity curve analysis to account for this. This involves monitoring intensity changes over time and adjusting for dilution by scaling the intensity with the peak concentration *I*_max_ at each pixel or voxel over time.

To facilitate comparisons between the MR Imaging datasets acquired under different parameters and anatomical areas, we normalized the slope CA_si_ for each pixel/voxel with the overall maximum dispersive slope values to obtain CA_si_^norm^.

Clinically, the rate of signal increase is linked to the steepness of CA wash-in and may reflect whether the CA is traveling through a singular shunting vessel (narrow histogram) or diffusing into multiple venous outflow paths (broader histogram) [27].

The spatial distribution of the above diagnostic parameters is displayed as two-dimensional or three-dimensional colormaps, with values assigned to corresponding pixels or voxels. The statistical distribution of the parameters is displayed by histograms.

### Statistical Analysis

For the in vitro validation, we compared the histogram distributions of CA_ToA_^norm^ and CA_si_^norm^ for DSA and GRASP through descriptive statistics (mean, standard deviation) and *χ*^2^ test. We calculated skewness, mean, median, and kurtosis for the histograms of in vivo type II, IV pAVMs, and CV-AVM (Tables 4 and 5).

**Table 4:** Metrics for CA_ToA_

ID	Skewness	Mean	Median	Kurtosis
ID001	2.24	4.40	4.59	16.90
ID002	3.12	6.80	3.75	12.70
ID003	6.252	11.12	10.25	51.29
ID006	3.62	5.76	4.51	32.12
ID008	2.04	14.54	10.20	7.56
ID009	2.86	18.56	15.64	13.12
ID012	0.92	44.90	33.81	2.50
ID020	0.95	19.80	17.43	4.18

**Table 5:** Metrics for CA_si_^norm^

ID	Skewness	Mean	Median	Kurtosis
ID001	− 0.12	0.46	0.45	4.00
ID002	0.76	0.42	0.39	3.00
ID003	0.30	0.57	0.53	2.19
ID006	0.69	0.36	0.31	2.71
ID008	0.17	0.48	0.46	2.17
ID009	− 0.18	0.43	0.45	2.70
ID012	0.27	0.49	0.50	2.70
ID020	− 0.16	0.50	0.51	2.44

## Results

### In Vitro Validation

**Fig. 2:**
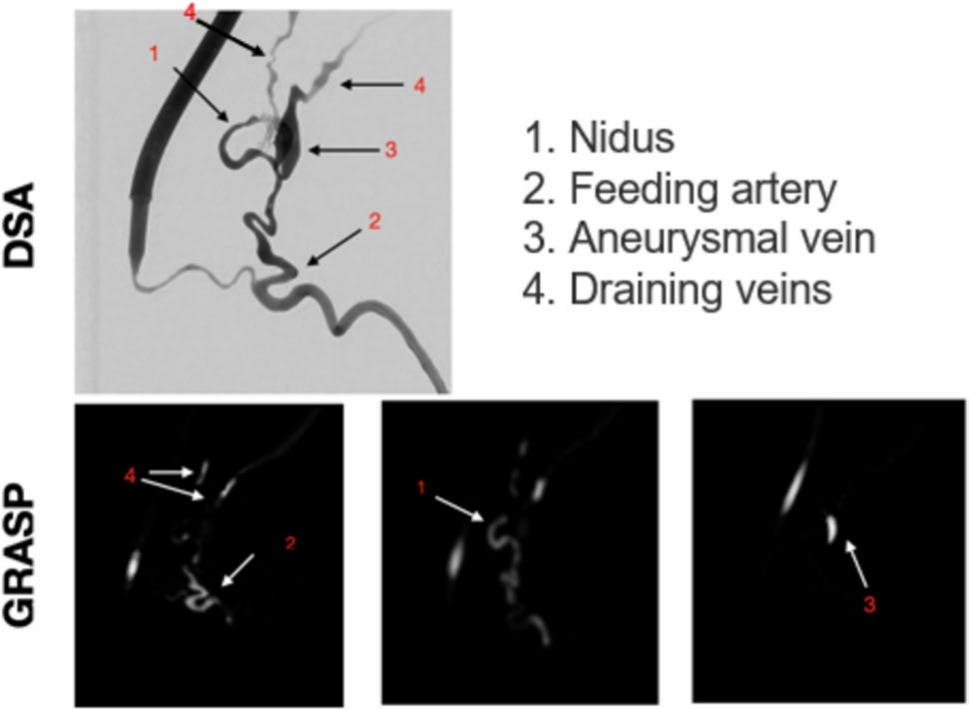
Selected DSA image and selected slices for GRASP at one time frame, highlighting the main features of the pAVM  [30, 36]

Figure 2 shows phantom DSA images (*t* = 5 s from the start of acquisition) and selected slices from GRASP imaging (*t* = 96.57 s from the start of acquisition). DSA images provide a sharper contrast of blood vessels and a higher spatial resolution. In MR Imaging, one of the two draining veins is not resolved in two points since it is smaller than the spatial resolution. Video 1 shows the earlier CA arrival using DSA compared to GRASP resulting from the smaller distance between the phantom and the injection point for DSA compared to GRASP.

**Fig. 3:**
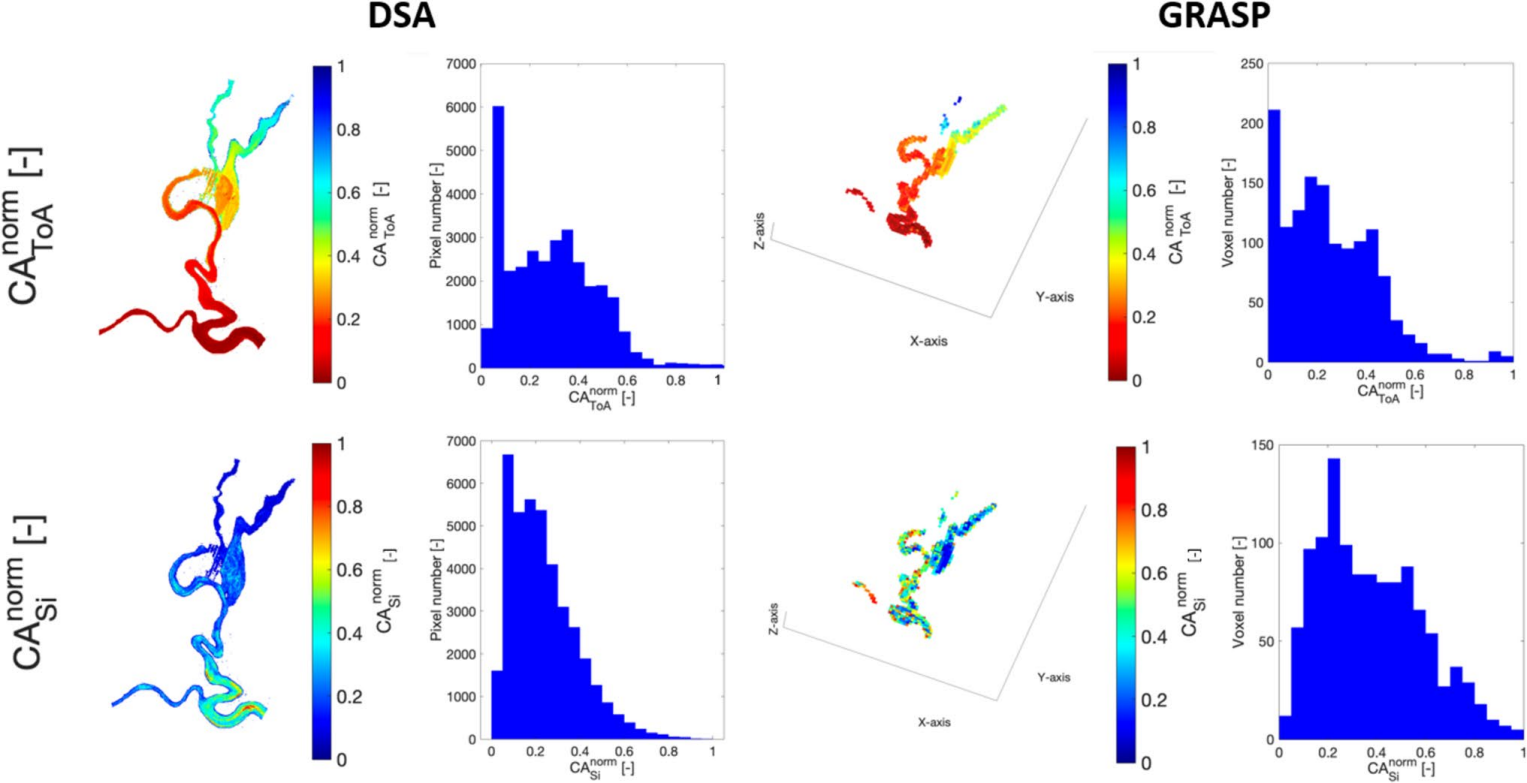
In vitro results. Spatial distribution and histogram representation of CA_ToA_^norm^ (first row) and CA_si_^norm^ (second row) obtained from DSA (left column) and GRASP (right column)

Colormaps and associated histograms of CA_ToA_^norm^ and CA_si_^norm^ for the phantom are reported in Fig. 3. The mean value of CA_ToA_^norm^ histogram distribution derived from MR imaging is slightly shifted to the left (average = 0.25, std = 0.18) compared to that derived from DSA (average = 0.28, std = 0.24). Despite normalization, the histograms cannot be identical since the 3D case accounts for the extension of the vessels in the third dimension, which gives a significant difference compared to the 2D case. We observe a decrease in the CA_ToA_^norm^ histogram distribution at 0.1–0.2 in both imaging modalities, which may represent the low filling of the aneurysmal vein, as observed in Frey et al. [27], but we do not see a second peak in the same histogram distribution representing the veins, due to their small volume.

The histogram of CA_si_^norm^ for GRASP reflects the more dispersed bolus for MR Imaging (average = 0.38, std = 0.21) compared to DSA (average = 0.28, std = 0.17). This likely reflects the greater distance between the phantom and the injection point of CA in MR Imaging compared to DSA. However, the *χ*^2^ test confirms that there is no statistically significant difference between MR-based data and DSA-based data (*χ*^2^ = 0.20, *p* = 0.65 for CA_ToA_^norm^ and *χ*^2^ = 0.21, *p* = 0.65 for CA_si_^norm^). It can thus be concluded that the histogram distributions show comparable trends.

### In Vivo Results

A representative 3D colormap of CA_ToA_ for ID005 is shown in Video 2. The 3D view allows a proper localization of the lesion: the *nidus* appears on the dorsal part of the hand as seen from DSA images, while it appears on the palmar part on the hand when looking at the MR-derived colormap. A figure in the Supplementary Material (Supplementary Material 1) summarizes all histograms of CA_ToA_ and CA_si_^norm^ and grouped by type of AVM. In the following sections, each AVM type is separately discussed.

#### Type II AVM

**Fig. 4:**
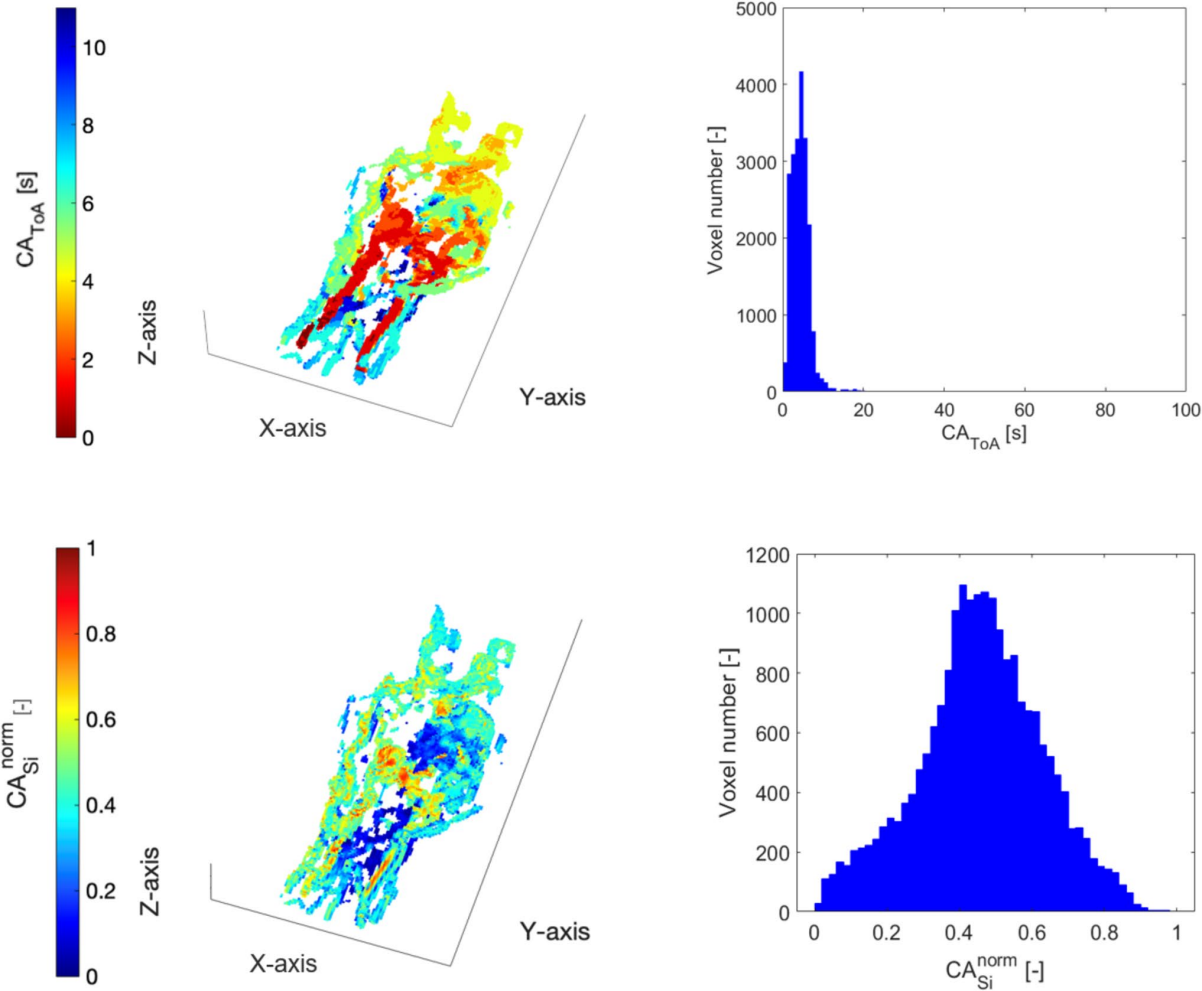
Type II pAVM, ID001. Spatial distribution (left) and histogram representation (right) of CA_ToA_ (first row) and CA_si_^norm^ (second row) obtained from MR images

Figure 4 displays the spatial distributions and associated histograms of CA_ToA_ and CA_si_^norm^ for a type II pAVM (ID001), which anatomically connects multiple arteries or arterioles to a *nidus* with multiple draining veins. The vessels colored red indicate areas with low CA_ToA_ (representing feeding arteries and high-flow arterio-venous shunts), and vessels colored blue represent areas with high CA_ToA_ (representing draining veins). The *nidus* is composed of more vessels than feeding arteries and draining veins, which results in a single peak in the CA_ToA_ histogram corresponding to all intermediate CA_ToA_ values in the *nidus*.

The CA_ToA_ histogram exhibits a right-skewed trend, with a single peak at 7 s, a steep decrease, and a short tail, indicating a fast shunting of the lesion with slow wash out. In the central area of the hand, vessels are not distinguishable, and toward the fingers, large cloudy areas are characterized by various CA_ToA_ values, representing fingers 2 and 3 involved in the lesion. The areas of the colormap that display high CA_ToA_ values are also characterized by higher CA_si_^norm^ values (range between 0.7 and 0.9), which is expected as arteries have higher blood velocities.

The CA_ToA_ values (Table 4) for ID001 and ID002 both exhibit low skewness values, indicating a moderate right-skewed distribution of CA_ToA_. In contrast, ID003 shows a higher skewness, reflecting a pronounced right tail, which indicates the presence of more extreme values in the data. The lower temporal resolution of ID003 might be the reason for a higher CA_ToA_ mean and median, which results in metrics more similar to CV-AVM patients. ID003 also has higher median and mean for CA_si_^norm^ distribution (Table 5) compared to ID001 and ID002, likely due to the low temporal resolution of the former.

#### Type IIIB AVM

**Fig. 5:**
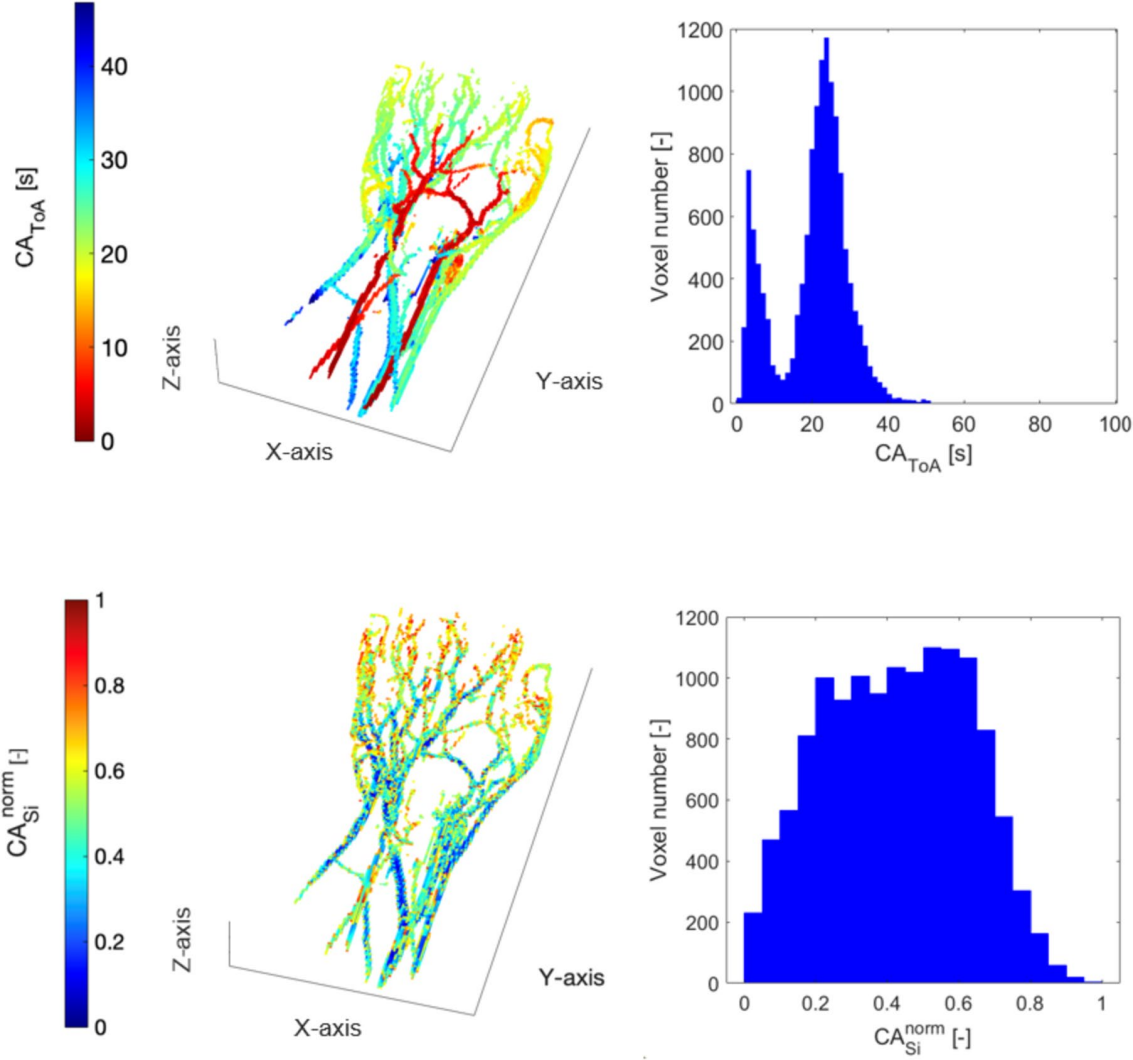
Type IIIB pAVM, ID004. Spatial distribution (left) and histogram representation (right) of CA_ToA_ (first row) and CA_si_^norm^ (second row) obtained from MR images. The first peak in the histogram distribution represents the arteries, the second the veins. The local minimum in the histogram distribution represents the low filling of the aneurysm

Figure 5 illustrates a type IIIB pAVM (ID004), which is characterized by multiple arteries joining an aneurysmally dilated vein followed by multiple draining veins. The histogram distribution details the circulation in the depicted hand, showing the arterial peak occurring at around 5 s and the broader venous peak at 25 s. The double-peak pattern indicates different hemodynamic characteristics compared to type II, as noted in a previous study by Frey et al., summarized in [5]. This pattern arises from the slower blood flow transition from artery to vein in type IIIB pAVMs, which is attributed to blood retention within the aneurysmal vein and its low filling. The broader venous peak compared to the arterial one is attributed to the large number of interconnected veins before CA reaches the large systemic veins and reflects the highly dispersive venous phase, which is typical of, e.g., type IIIb morphologies that may include multiple, dilated, draining veins.

**Fig. 6:**
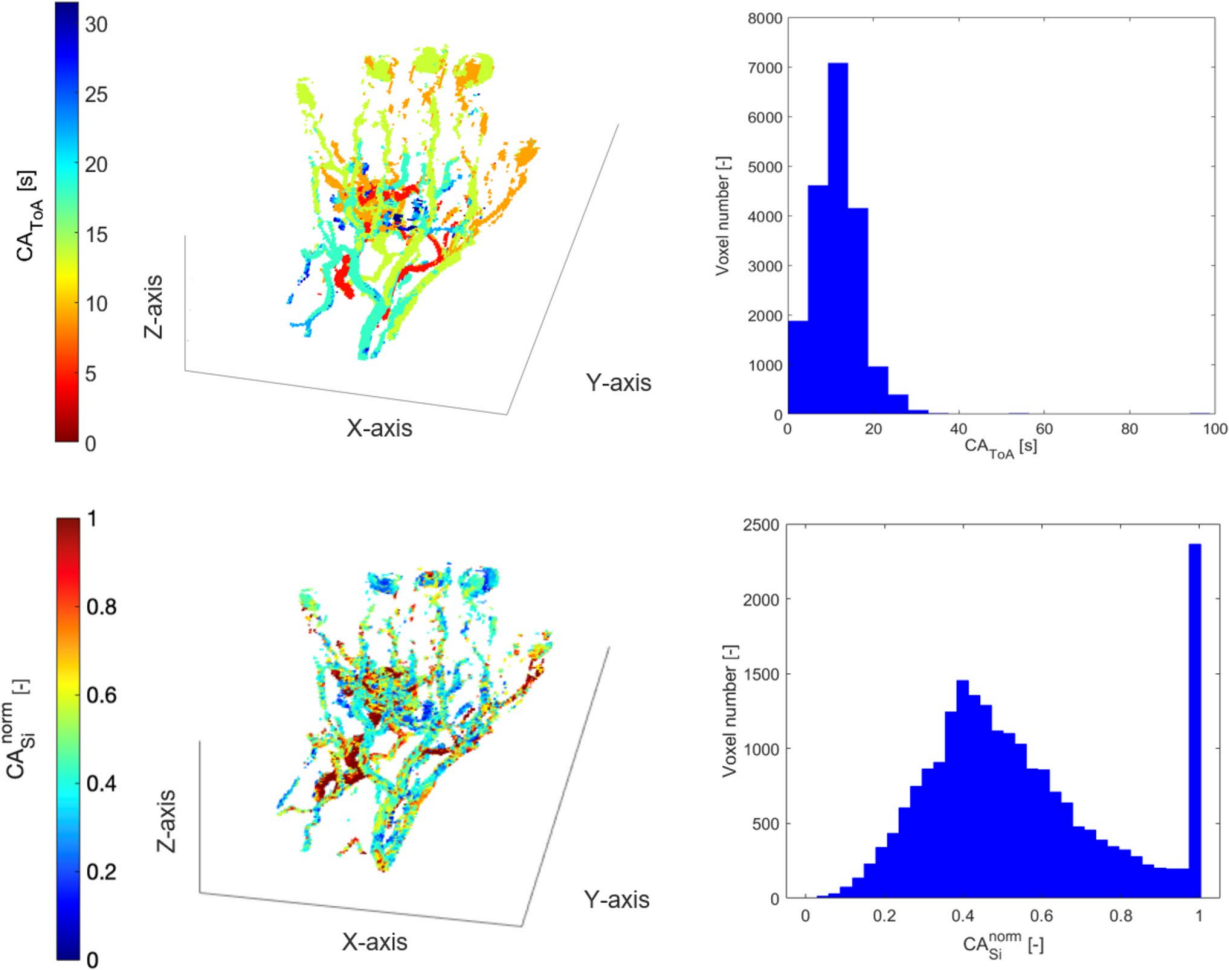
Type IIIB pAVM, ID005. Spatial distribution (left) and histogram representation (right) of CA_ToA_ (first row) and CA_si_^norm^ (second row) obtained from MR images

In contrast, Fig. 6 shows a CA_ToA_ histogram (ID005 with a type IIIB pAVM) with a single peak at 15 s. The absence of the double-peak pattern is likely due to a low temporal resolution, which clusters the bins in a few different values.

Video 2 illustrates a 3D colormap of CA_ToA_ derived from a CE-trMRA dataset for ID005 (selected patient case) and its comparison with DSA. It highlights the importance of having a 3D mapping of the lesion: the malformation seems to be on the dorsal part of the image on DSA, while it is clear from the 3D colormap derived from CE-trMRA that it is on the palmar part of the hand.

The CA_si_^norm^ histogram of ID004 exhibits a wide bell shape. On the contrary, a peak at CA_si_^norm^ = 1 of the histogram is observed in ID005 due to the low temporal resolution, which makes the main feeding artery fill in one time step.

#### Type IV AVM

**Fig. 7:**
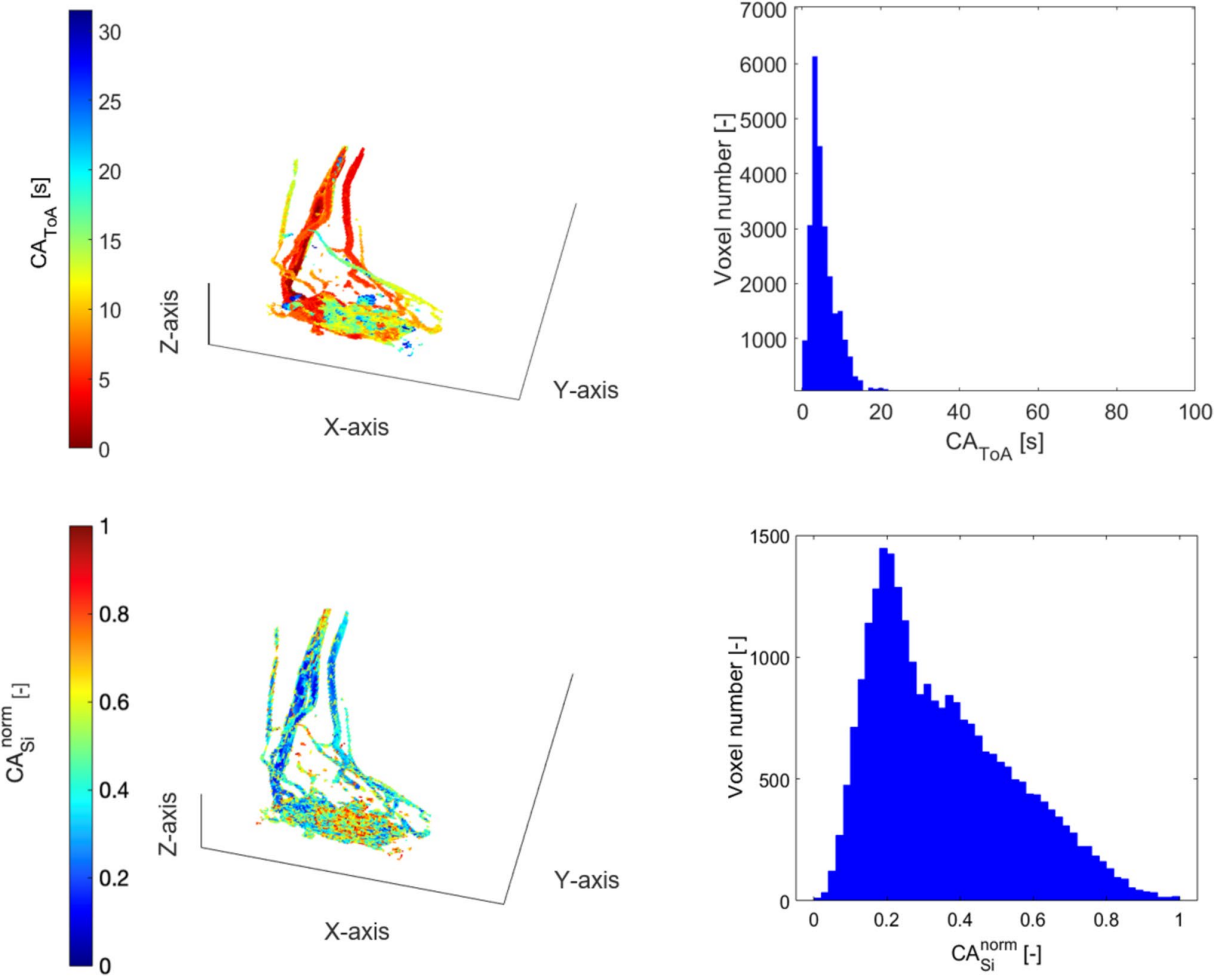
Type IV pAVM, ID006. Spatial distribution (left) and histogram representation (right) of CA_ToA_ (first row) and CA_si_^norm^ (second row) obtained from MR images

Figure 7 displays a type IV pAVM (ID006), which is characterized by numerous small arterio-venous shunts. The colormap shows a complex and nebulous pattern in the central area of the foot, characterized by values of CA_ToA_ between 8 and 12 s. The histogram aligns with the colormap’s pattern, exhibiting a right-skewed trend with a peak at 4 s. The CA_ToA_ trend is similar to type II pAVMs but shifted toward higher values, attributed to the shunt regions with numerous vessels exhibiting intermediate CA_ToA_. This trend aligns with the pattern from DSA images of the same pAVMs published previously [5] showing higher CA_ToA_ values than type II. The CA_si_^norm^ pattern is right-skewed, with an average value of 0.2 and characterized by a slow and linear decrease.

The metrics of ID020 for CA_ToA_ (Table 4) reveal that type IV pAVMs demonstrate a distinct pattern in CA_ToA_ compared to those in type II. The higher mean and median values suggest slower dynamics, and the moderate skewness value suggests some moderate CA_ToA_. The kurtosis values indicate that while ID020 has a relatively moderate distribution, ID001 and ID002 exhibit significant variability with a tendency for extreme values, contrasting with ID003’s flatter distribution.

Regarding the metrics of CA_si_^norm^ (Table 5), the most distinguishing feature of ID020 lies in its skewness and the fact that it exhibits a slight left-skew compared to the positive skewness seen in ID002 and ID003. The kurtosis indicates that while ID020 has a peaked distribution, it does not exhibit extreme values, as in ID001 and ID002.

#### Type CV-AVM

**Fig. 8:**
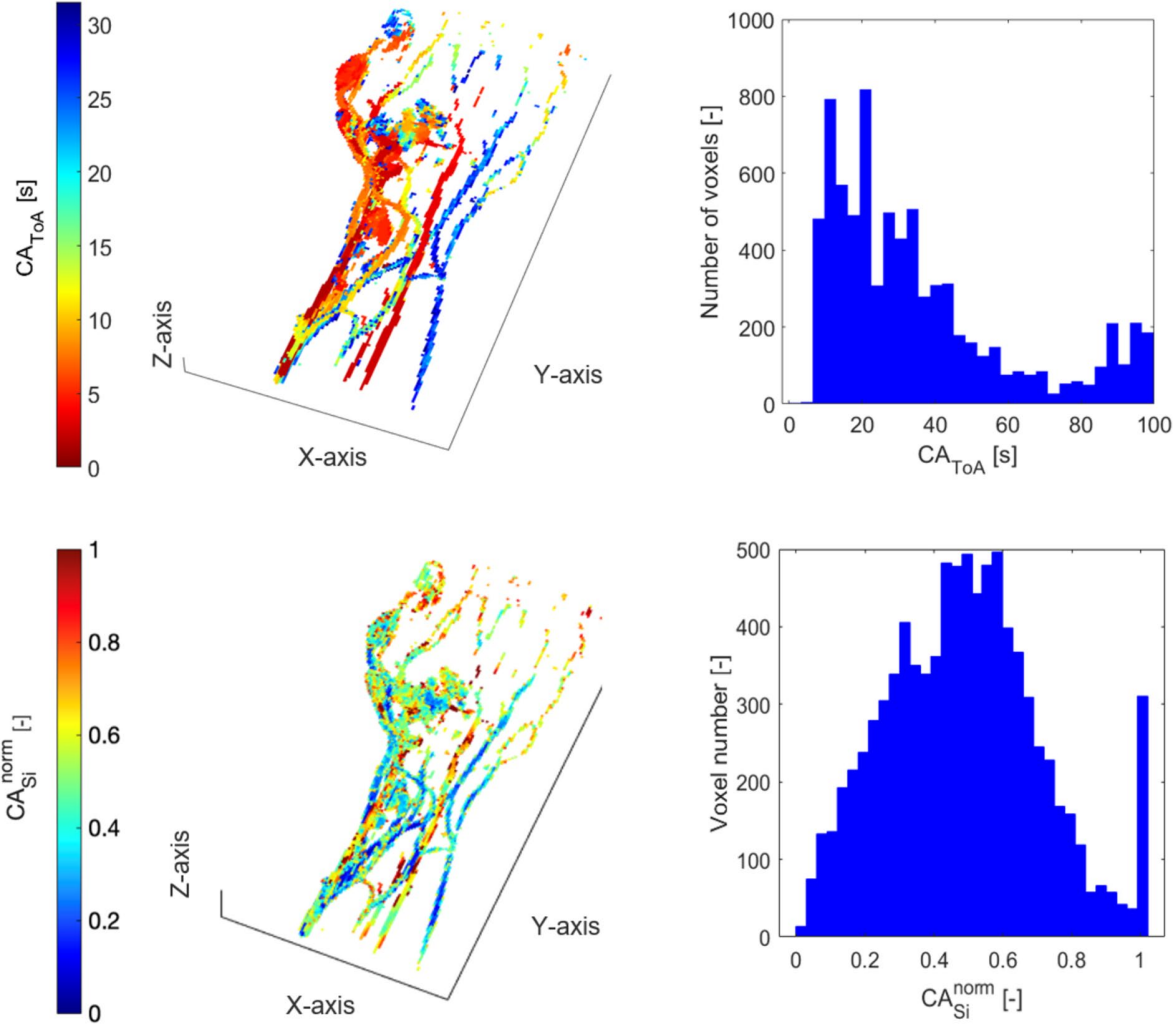
CV-AVM, ID012. Spatial distribution (left) and histogram representation (right) of CA_ToA_ (first row) and CA_si_^norm^ (second row) obtained from MR images

Figure 8 shows a CV-AVM, another type of arterio-venous shunting of even smaller diameter of vessels—between capillaries and venules. The colormap of CA_ToA_ exhibits a wide range of values spanning from red to blue. The histogram shows a higher maximum CA_ToA_ than all the other pAVM types, with values reaching more than 100 s. The histogram pattern exhibits an initial peak around 12 s; then it decreases gradually up to 70 s while exhibiting a right-skewness. Subsequently, a second section shows an area of high CA_ToA_. The longer acquisition times of CE-trMRA allow us to observe a tail of values, which likely represents delayed venous return.

The CA_ToA_ distribution for patients diagnosed with CV-AVM varies, with the first peak generally appearing less right-skewed compared to type IV malformations. CV-AVM patients are distinguished by their higher mean and median values of CA_ToA_ (Table 4) compared to other pAVM types, reflecting a broader and skewed distribution toward higher values. The skewed nature toward higher values reflects the broader spread and highlights the presence of higher central values in CV-AVMs. The CA_si_^norm^ metrics for CV-AVMs (Table 5) show mild skewness with a slight negative or positive trend. Kurtosis values varying between 2.40 and 2.70 indicate normal distributions with light tails. The mean and median values are quite consistent, which suggests symmetric distributions.

Generally, types II and III pAVMs show more variability in the distributions of CA_si_^norm^, while CV-AVMs and type IV pAVMs exhibit more uniform and symmetric distributions.

## Discussion

In this work, we present a set of diagnostic parameters indicative of the type of pAVMs, which can be measured from MR Imaging. The study is divided into two parts. The in vitro part serves as a validation of the hemodynamic markers previously computed [27] and characterizes pAVMs in DSA and MR environments. The in vivo part investigates the feasibility of characterizing hemodynamic parameters of in vivo pAVMs through CE-trMRA datasets and explores the clinical advantages of using MR Imaging compared to DSA. This characterization is achieved by analyzing the CA transport parameters, i.e., by computing the time required for CA to appear at a certain location (CA_ToA_) and the local rate of CA-signal increase (CA_si_^norm^).

### In Vitro Validation

We validated the proposed hemodynamic markers using controlled in vitro experiments comparing DSA and MR Imaging in a 3D-printed phantom. The CA_ToA_^norm^ histogram from MR Imaging (mean = 0.25, std = 0.18) closely resembled that from DSA (mean = 0.28, std = 0.24). Similarly, CA_si_^norm^ from MR Imaging (mean = 0.38, std = 0.21) showed dispersion patterns comparable to DSA (mean = 0.28, std = 0.17) and reflects the effect of different injection protocols. Although CA_si_^norm^ is well suited to characterize the dispersivity of the lesions, this hemodynamic marker is strongly dependent on the injection protocol. Hence, the broader histogram of CA_si_^norm^ derived from MR Imaging compared to DSA is expected as a result of the larger distance between injection point and phantom that mimics the intra-arterial injection in DSA and the intra-venous injection in MR Imaging environments. Despite differences in acquisition setups, the *χ*^2^ test confirmed no statistically significant differences in histogram distributions (CA_ToA_^norm^: *χ*^2^ = 0.20, *p* = 0.65; CA_si_^norm^: *χ*^2^ = 0.21, *p* = 0.65), validating the transferability of the hemodynamic markers to MR-based classification.

The validation of the proposed pAVM hemodynamic markers [27] to characterize pAVMs through controlled experiments, MR Imaging and DSA, provides, to the best of our knowledge, the first experimental set-up of a detailed model of a pAVM with fully known geometry of the lesion and surrounding vasculature, using clinical imaging modalities. This validation establishes a foundation for its application into clinical practice, where it can be used to analyze blood flow dynamics and to develop new diagnostic and therapeutic markers, eventually to optimize treatment strategies and improve patient outcomes.

### Application of the Metrics to In Vivo Data

#### Time of Arrival (CA_ToA_)

The image processing on the in vivo MR image datasets of extremities shows that CA_ToA_ is a powerful hemodynamic parameter for the classification of pAVMs. The histogram shapes of CA_ToA_ are able to capture local blood flow velocities, shunting behaviors, and hemodynamic characteristics of the pAVM types.

We found that the spatial distributions (colormaps) and histogram representations for CA_ToA_ with sufficiently high MR acquisition parameters align with trends previously observed computationally and DSA recordings [5]. Interestingly, MR Imaging is able to distinguish CV-AVMs by the presence of peaks in the CA_ToA_ histogram around 100 s, a feature which would not be extracted from DSA images where venous return visualization is limited, and scans often end prematurely to limit radiation exposure. This suggests that late CA_ToA_ could be a clinically relevant indicator for identifying CV-AVMs among other pAVM types.

For clinicians, knowledge of the distribution of CA_ToA_ in the lesion and the surrounding vessels provides an indication of the types of blood vessels affected by the malformation: late arrival times suggest the presence of microvascular pAVMs with higher resistance than lesions in large vessels that exhibit early arrival times. In addition, distinguishing between normalized and non-normalized CA_ToA_ is crucial for clinicians. While normalized CA_ToA_ spatial distributions and histograms allow for comparative analysis across different images, non-normalized CA_ToA_ colormaps and histograms provide clinicians with precise temporal information to identify blood flow abnormalities. Such information can be an indicator of the lesion size, the role of the lesion within other vascular structures, and hence used to monitor treatment progress.

#### Dispersive Slope (CA_si_)

The right-skewed histogram and higher dispersive upslope values of microcirculatory type IV pAVM compared to types II and III may primarily arise from the extensive network of shunting vessels and pathological connections with low dispersion areas characteristic of type IV, resulting in lower dispersive slope values and shifting the histogram toward lower CA_si_^norm^ values.

Interestingly, CV-AVMs do not exhibit a consistent trend in the CA_si_^norm^ histograms computed from CE-trMRA. While greater dispersion and low CA_si_^norm^ values might be expected due to microcirculation, this is not observed, likely due to the absence of a *nidus* and the limited spatial resolution of CE-trMRA.

### General Considerations

We have shown that our MR-based datasets with a temporal resolution around 1 s can provide a detailed characterization of CA distribution patterns and dynamics and provide sufficient temporal detail to distinguish between different pAVM types, supporting lesion characterization.

MR-based datasets with lower temporal resolution cannot capture important hemodynamic information and do not show an accurate histogram classification. For example, for patients ID005 and ID012, we see that in the CA_si_^norm^, there is a peak at unity, which represents the filling of the arteries in one time step. This observation does not hold for the CA_ToA_ of ID012 (CV-AVM) because we focus on the presence of late CA_ToA_, which is not affected by the temporal resolution.

Another acquisition parameter which has to be considered is the spatial resolution, crucial for precise vessel visualization. CE-trMRA typically has lower spatial resolution compared to DSA, leading to the inability to visualize small blood vessels and partial volume effects [44]. This can blur fine anatomical details, e.g., in CV-AVM and not allow the differentiation of adjacent small vessels as voxels blend blood vessels with the surrounding tissue. Slice thickness impacts the quality of 3D rendering too: smaller slice thickness reduces small gaps and stair-steps artifacts between slices and contributes to smoother 3D visualizations, producing a more continuous and detailed representation of vascular structures.

DSA has limitations despite its ability to visualize pAVM development over time. Firstly, it is invasive in its injection method, requiring a catheter for intravascular access. In contrast, even with a peripheral injection, our MR Imaging approach still yields high-quality histograms.

In addition, DSA provides a two-dimensional colormap due to single-projection imaging, which can miscalculate diagnostic parameters where veins and arteries overlap. This limitation hinders understanding of the three-dimensional angioarchitecture (location, size, and shape of the malformation to be treated), which is essential for accurate clinical diagnosis and treatment planning [49, 50]. The 3D colormaps provide diagnostic aids, allowing clinicians to have a quick 3D visualization of the pAVM morphology and facilitate arterio-venous separation, as shown by Schubert et al. [51] for cAVMs and efficient localization of hemodynamic hotspots (e.g., *nidus*) in the 3D space [52]. For instance, in ID001, the 3D colormap provides the physicians a quick understanding that fingers 1, 4, and 5 have poor perfusion due to a steal phenomenon.

Finally, the longer scan time of CE-trMRA compared to DSA may improve the detection of microvascular AVMs that would otherwise remain unnoticed on shorter DSA acquisitions. This could support more accurate lesion classification and improved treatment planning.

Our findings have a substantial impact on pAVM diagnosis, since they may allow an automated classification of lesions, solely based on the analysis of radiation-free MR images, which may reduce the number of misdiagnoses of pAVMs and may improve personalized treatment. Although we did not perform a diagnostic accuracy or clinical utility study, this study clearly supports the use of MR imaging as primary tool for baseline diagnostics and follow-up. This recommendation aligns with the results reported in [53–55], which suggest that additional diagnostic DSA is not required for treatment planning prior to therapeutic endovascular procedures.

The proposed classification framework for pAVM can be adapted to characterize pAVMs of other anatomical areas. A different trade-off between spatial and temporal resolution might be established for different body regions. Using a radial sequence might be beneficial in terms of better vascular contrast, vessel sharpness, and motion robustness for pAVM in the body's abdominal region [56]. However, this is beyond the scope of the present study.

### Limitations

The present study has highlighted the use of MR Imaging extracted CA-based metrics for the classification of pAVMs. A relatively small number of datasets were employed, limited to a specific body anatomy. Furthermore, the study did not include type I and IIIa pAVMs because MR datasets were not available for these types.

Another limitation of the study is the low in-plane spatial resolution of the MR datasets that did not allow sub-millimeter vessels to be distinguished and relevant hemodynamic features to be identified. Further improvements in MR Imaging acquisition parameters might be required to fully replace DSA in diagnosing pAVMs.

Further work might be needed to fully assess and quantify the impact on injection on CA_si_^norm^ as studies show varying results between the injected GBCA volume and injection rate, and the concentration and the signal intensity [57–61]. Thus, our results for CA_si_^norm^ might be dependent on such injection parameters.

Additionally, the use of a finite difference approximation to compute the CA_si_^norm^ introduces sensitivity to the temporal resolution of the acquisition. Low temporal resolution may result in underestimation or oversimplification of the true CA dynamics, especially in vessels where contrast changes rapidly. This should be considered when interpreting and comparing CA_si_^norm^ metrics across datasets with different acquisition settings.

### Conclusions

We have shown that MR Imaging allows the estimation of CA transport parameters such as CA_ToA_ and CA_si_^norm^ in both in vitro and in vivo datasets, allowing to characterize different types of pAVMs without the need of DSA. The characterization of pAVMs through such hemodynamic markers has the potential to become a diagnostic procedure for the surgical navigation and classification of pAVMs for clinicians. In the future, this approach could enhance diagnostic precision, guide the development of more effective treatment strategies, and help reduce the complication rates and the incidence of misdiagnosed cases.

## Supplementary Information

Below is the link to the electronic supplementary material.

Supplementary file1 (PNG 76 kb) **Supplementary Material 1.** The image summarizes the CA_ToA_ and CA_si_^norm^ histograms derived from MR Imaging for all patients included in the study, grouped by type.

Supplementary file2 (MOV 7814 kb) **Video 1**. The movie shows the arrival of the CA in the phantom, in DSA (left) and GRASP (right). We can observe that in DSA the CA arrives earlier, due to the closer injection compared to MRI.

Supplementary file3 (MOV 2967 kb) **Video 2**. The animated image reports the 2D colormap of CA_ToA_ derived from DSA (left) and the 3D colormap (.gif) (right) derived from MR Imaging, of ID005. We can observe that the 3D colormap allows a 3D localization of the hemodynamic features of the pAVM: we can localize the *nidus* of the malformation on the palmar part of the hand, rather than on the dorsal part of the hand, as it appears in 2D DSA.

## Abbreviations

*A*: Absorbance
AVM: Arterio-venous malformation
CA: Contrast agent
CA_ToA_: Time of arrival of the contrast agent
CA_ToA_^norm^: Normalized time of arrival of the contrast agent
CA_si_: Dispersive slope of the contrast agent
CA_si_^norm^: Normalized dispersive slope of the contrast agent
CE-trMRA: Contrast-enhanced time resolved magnetic resonance angiography
CT: Computed tomography
CV-AVM: Capillary–venulous arterio-venous malformation
CVM: Congenital vascular malformation
DSA: Digital subtraction angiography
GRASP: Golden-angle radial sparse parallel
GBCA: Gadolinium-based contrast agent
*I*_CA,max_: Peak contrast agent concentration
MR: Magnetic resonance
*Q*: Flow rate
ToA: Time of arrival
US: Ultrasound
*V*: Volume
