# Supplementary figures and images for: Hemodynamic Characterization of Peripheral Arterio-Venous Malformations Using Rapid Contrast-Enhanced MR Imaging: An In Vitro and In Vivo Study
Source: Ann Biomed Eng. 2025 Jun 13;53(9):2147–63. doi: 10.1007/s10439-025-03766-3 (PMC12390877; doi:10.1007/s10439-025-03766-3)

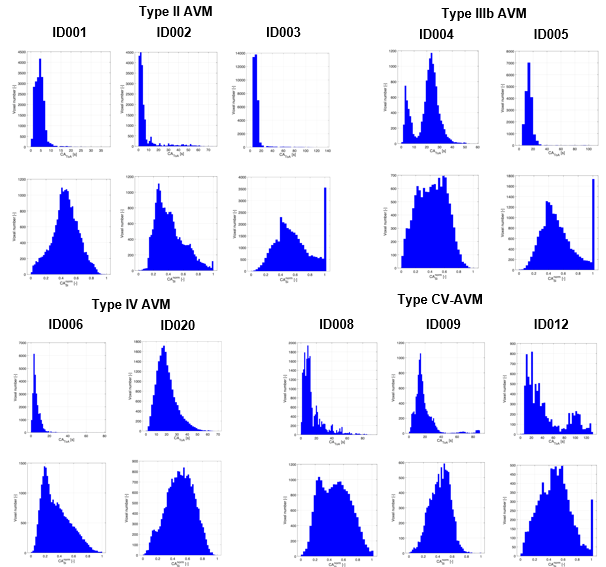

Supplement: Supplementary file 1 — Supplementary file1 (PNG 76 kb) Supplementary Material 1. The image summarizes the CAToA and CAsinorm histograms derived from MR Imaging for all patients included in the study, grouped by type. [file 10439_2025_3766_MOESM1_ESM.png]
